# Acidity enhancement through synergy of penta- and tetra-coordinated aluminum species in amorphous silica networks

**DOI:** 10.1038/s41467-019-13907-7

**Published:** 2020-01-13

**Authors:** Zichun Wang, Tong Li, Yijiao Jiang, Olivier Lafon, Zongwen Liu, Julien Trébosc, Alfons Baiker, Jean-Paul Amoureux, Jun Huang

**Affiliations:** 1 Laboratory for Catalysis Engineering, School of Chemical and Biomolecular Engineering & Sydney Nano Institute, The University of Sydney, Sydney, NSW 2006 Australia; 2 Department of Engineering, Macquarie University, Sydney, NSW 2109 Australia; 3 Institute for Materials & ZGH, Ruhr-Universität Bochum, 44801 Bochum, Germany; 4 Univ. Lille, CNRS, UMR 8181, UCCS-Unité de Catalyse et de Chimie du Solide, F-59000 Lille, France; 5 Institut Universitaire de France, Centrale Lille, ENSCL, Villeneuve-d’Ascq, France; 6 Univ. Lille, CNRS, INRA, Centrale Lille, ENSCL, Univ. Artois, FR 2638 - IMEC - Institut Michel-Eugène Chevreul, F-59000 Lille, France; 7 Institute for Chemical and Bioengineering, Department of Chemistry and Applied Biosciences, ETH, Zürich, Hönggerberg, HCI CH-8093 Switzerland; 8 Bruker Biospin, 34, rue de l’industrie, 67166 Wissembourg, France; 9 Riken NMR Science and Development Division, Yokohama, 230-0045 Kanagawa Japan

**Keywords:** Catalytic mechanisms, Heterogeneous catalysis, Chemical engineering

## Abstract

Amorphous silica-aluminas (ASAs) are widely used in acid-catalyzed C-H activation reactions and biomass conversions in large scale, which can be promoted by increasing the strength of surface Brønsted acid sites (BAS). Here, we demonstrate the first observation on a synergistic effect caused by two neighboring Al centers interacting with the same silanol group in flame-made ASAs with high Al content. The two close Al centers decrease the electron density on the silanol oxygen and thereby enhance its acidity, which is comparable to that of dealuminated zeolites, while ASAs with small or moderate Al contents provide mainly moderate acidity, much lower than that of zeolites. The ASAs with enhanced acidity exhibit outstanding performances in C–H bond activation of benzene and glucose dehydration to 5-hydroxymethylfurfural, simultaneously with an excellent calcination stability and resistance to leaching, and they offer an interesting potential for a wide range of acid and multifunctional catalysis.

## Abstract (web summary)

Increasing the strength of silanols in silica-aluminas can promote their catalytic activity in various applications. Here, the authors present a synergistic effect of two neighboring Al sites on the same silanol group, which can enhance the strength of silanol proton from moderate to zeolitic strength.

## Introduction

Silica-alumina materials, particularly crystalline zeolites and amorphous silica-aluminas (ASAs), are among the most popular solid acids that have been widely commercialized as efficient and environmentally friendly catalysts in the petrochemical industry^1^, and in bio-refinery^2^. These materials can provide Brønsted acid sites (BAS) with tunable density and strength, which facilitates the optimization of the surface acidity to promote a series of important industrial chemical reactions, through the formation of surface complexes or transition states by proton transfer from BAS to reactants^3–6^, such as to initialize C–H activation for hydrocarbon conversions^7–12^. Zeolites with strong Brønsted acidity, are of increasing importance in various sustainable processes, in the fields of biomass conversion, CO_2_ capture and conversion, air-pollution remediation, and water purification^13^. For instance, zeolites can efficiently catalyze the redox disproportionative conversion of biomass-derived sugars into α-hydroxy acids^14^, and they are more active than ASA catalysts, albeit the latter facilitate improved molecular diffusion in the porous network^15^. The lower performance of ASA in many catalytic applications is widely attributed to their moderate Brønsted acidity^1^, which fostered recent works on the discovery of ASAs with increased Brønsted acidity^5^, aiming at expanding their applications in a broad range of fields.

The formation of BAS in silica-aluminas is based on aluminum centers distributed in the silica framework or network^6,16–20^, as (i) a tetra-coordinated aluminum species (Al^IV^), replacing a Si^4+^ atom in the zeolite framework, can introduce a negatively charged framework oxygen to be balanced by a proton, resulting in bridging OH groups^6,17,18^, and (ii) the interaction between Al^IV^ atoms and neighboring silanols in the silica network can provide acidic OH groups, acting as BAS in ASA^5,21–24^. In crystalline zeolites, it is well accepted that increasing the Si/Al ratio can enhance the BAS strength by increasing the overall electronegativity^25^. However, the amount of BAS (e.g. bridging SiOHAl groups) is then significantly reduced, owing to the fewer Al sites in the framework. Alternatively, the introduction of extra-framework cations, such as Al^3+^ and La^3+^, via dealumination or ion exchange can significantly improve the BAS strength due to a synergistic effect between Lewis acid sites (LAS) and nearby BAS^4,17^. Those solid acids have been widely applied in gas-phase cracking, such as fluid catalytic cracking processes^26^. However, extra-framework cations can easily leach out from solid acids during the liquid-phase reactions^27^. Additionally, the synergy between two nearby Al sites in the zeolite framework is impossible due to the absence of Al-O-Al linkage based on Löwenstein’s rule^28^.

Enhancing the BAS strength in ASAs still remains a significant challenge. Although a surface bridging SiOHAl model has been proposed for the formation of BAS on ASA^21,29^, the strength of the BAS on ASA is generally lower than that on crystalline zeolites^1^, since the amorphous structure of ASAs weakens the Al-O bonds (2.94–4.43 Å)^24^, compared to those in the crystalline zeolite framework (1.88–2.0 Å)^30^. Two models have been proposed to account for BAS generation in ASA: (i) a flexible coordination between the Al atom and the neighboring silanol oxygen atom^5,23^, and (ii) a pseudo-bridging silanol (PBS) with a nearby Al atom^24,31,32^. In both models, it was proposed that a Lewis acidic Al center interacts with a nearby silanol group, withdrawing electron density from the hydroxyl O atom to enhance the acid strength of the hydroxyl proton. Notably, these models mainly account for the formation of moderate BAS. Nevertheless, the presence of an additional Al species in the vicinity of the SiOH site may further enhance the acid strength of ASA via a synergistic effect, which has not yet been evidenced to the best of our knowledge.

In this work, the synergy between Al species in the ASA network has been studied using solid-state NMR spectroscopy and atom probe tomography (APT). The ^27^Al double-quantum single-quantum (DQ-SQ) through-space homonuclear correlation (D-HOMCOR) NMR experiments allow us to probe ^27^Al-^27^Al proximities by applying recoupling sequences that restore the dipolar interaction between neighboring ^27^Al spins^33–36^. Unlike for crystallized materials, the investigation of the location and distribution of Al atoms is impossible in ASAs by routine characterization methods, such as X-ray diffraction (XRD) and high-resolution transmission electron microscopy (HRTEM), due to their amorphous structure. Atom probe tomography (APT) can provide quantitative three-dimensional (3D) information on elemental distributions in catalyst nanoparticles at the atomic scale^37,38^. It has recently been employed with sub-nanometer-scale resolution on zeolite-based catalysts to establish their structure–composition–property relationships^39–43^. Here, we apply APT for the same purpose in ASAs^44–47^. The combined investigations of APT on Al distribution and ^27^Al and ^1^H DQ-SQ NMR experiments reveal, for the first time, the existence of a synergy between Al species in the ASA network. This synergy can significantly enhance the acid strength of ASA, as demonstrated by the H/D exchange with deuterated benzene. The beneficial effect of the enhanced acidity is demonstrated by an example of the liquid-phase catalytic dehydration of glucose to 5-hydroxymethylfurfural, which is an important building block in the production of various valuable chemicals, such as liquid alkanes, biofuels, and furan derivatives^48^.

## Results

### Local structure of SA/10

The ASA materials were prepared by flame spray pyrolysis as described in Supplementary Methods and they are designated as SA/x, where x = 10 or 50 represents the percentage of Al atoms with respect to the total amount of Al and Si atoms in the precursor. APT has been applied to show the distribution of Al within the SA/10 as shown in Supplementary Fig. 1 and Supplementary Movie 1 in the Supplementary Information. The tomographic reconstruction qualitatively shows a homogeneous distribution of Al, Si and O (Supplementary Fig. 1b, c, d), where each sphere represents the 3D position of an individual atom. In agreement with recent energy-dispersive X-ray atom mapping investigations^49^, the APT reveals a homogeneous dispersion of Al species in the silica network, similar to that observed in well-developed crystalline zeolites^42,43^. Due to the lack of evidence for the existence of zeolite-like bridging OH groups in ASA, Al atoms in SA/10 can be expected either bridged to SiO (SiOAl) or located nearby the silanol groups (SiO(H)∙∙∙Al), as reported in literature^16,21^.

**Fig. 1:**
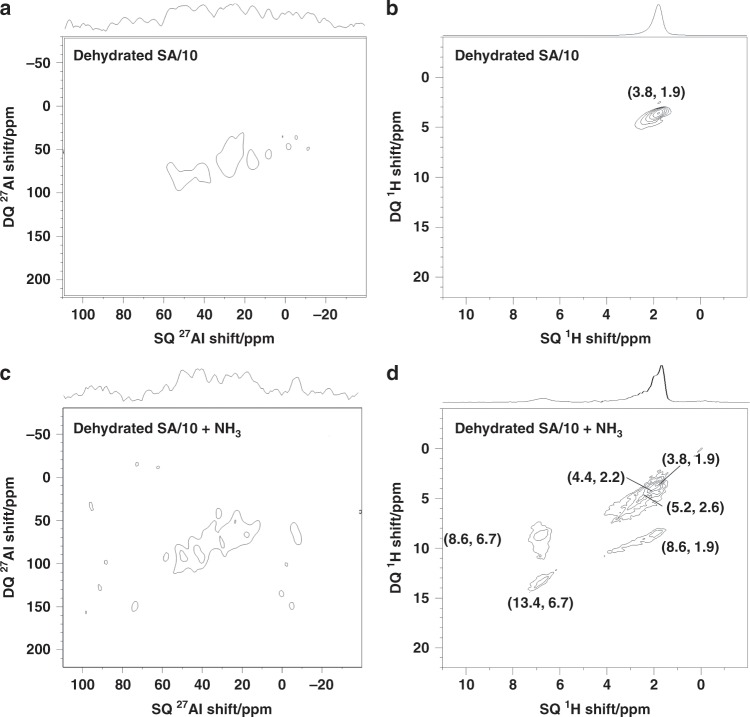
DQ-SQ *D*-HOMCOR 2D NMR spectra of SA/10. The spectra of subfigures **a**, **c** and **b**, **d** are for ^27^Al and ^1^H NMR spectra, respectively, acquired at 18.8 T with a MAS frequency of *ν*_R_ = 20 kHz. The spectra of subfigures **a** and **b** were acquired for SA/10 dehydrated at 723 K for 12 h under vacuum, whereas the spectra of subfigures **c** and **d** were dehydrated and loaded with ammonia, and then evacuated at 393 K for 1 h to remove the weakly physisorbed molecules.

Supplementary Fig. 2a displays the single-pulse 1D ^27^Al MAS spectrum of SA/10. It exhibits three peaks at 50, 30, and 4 ppm, assigned to tetra- (Al^IV^), penta- (Al^V^) and hexa- (Al^VI^) coordinated Al sites, respectively^16,50^. In the DQ-SQ 2D spectra, as shown in Fig. 1, autocorrelation diagonal peaks resonating at the frequencies (2*ν*, *ν*) along the indirect and direct dimensions, respectively, indicate the proximities between nuclei with identical isotropic shifts, *ν*, whereas the cross-peak pairs resonating at frequencies (*ν*_a_ + *ν*_b_, *ν*_a_) and (*ν*_a_ + *ν*_b_, *ν*_b_) specify proximities between nuclei resonating at distinct frequencies, *ν*_a_ and *ν*_b_, in the 1D spectra. The ^27^Al DQ-SQ 2D spectrum (Fig. 1a) exhibits a weak diagonal peak at (60, 30) ppm, which indicates proximities between Al^V^ sites in SA/10. No other obvious ^27^Al correlation signal could be detected, which was confirmed by the ^27^Al slices that were extracted at the corresponding shifts of these correlation signals (Supplementary Fig. 2e–i). The ^1^H MAS spectrum of SA/10 shown in Supplementary Fig. 2b exhibits a single peak resonating at 1.9 ppm, which is ascribed to the silanol protons. The ^1^H DQ-SQ 2D spectrum shows a single strong signal at (3.8, 1.9) ppm (Fig. 1b). This peak indicates the spatial proximity of many silanol groups on the surface.

The presence of BAS has been further confirmed by the adsorption of a basic molecular probe, ammonia. The ^1^H spectrum of SA/10 loaded with ammonia (see Supplementary Fig. 2d) exhibits a peak at *δ*_1H_ = 6.7 ppm, which is ascribed to ammonium ions^6^. The presence of an auto-correlation peak at (13.4, 6.7) ppm in the ^1^H DQ-SQ spectrum of Fig. 1d indicates that the ammonium protons are dipolar coupled and hence, do not exhibit isotropic motion in the sample. The cross-peaks at (8.6, 1.9) and (8.6, 6.7) ppm in the same spectrum demonstrate mainly that some ammonia ions stay adsorbed near the SiOH groups. The peak at *δ*_1H_ = 2.6 ppm is assigned to ammonia adsorbed at LAS^51–54^. The autocorrelation peak at (5.2, 2.6) ppm in Fig. 1d indicates that the protons of adsorbed ammonia are dipolar coupled and hence that these molecules do not exhibit isotropic motions. Conversely, the ^27^Al 1D (Supplementary Fig. 2c) and 2D *D*-HOMCOR (Fig. 1c) spectra are not significantly modified by the adsorption of ammonia. In particular, we only detect a weak Al^V^ diagonal peak in the 2D *D*-HOMCOR spectrum, and no other correlation signal could be detected as observed with dehydrated SA/10 (Fig. 1a).

The ^13^C NMR signal of SA/10 loaded with CH_3_^13^COCH_3_ probe molecule resonates at *δ*_13C_ = 213 ppm^5^. This value is similar to those observed in most ASA samples^22^, but is much smaller than that of zeolite H-ZSM-5 (*δ*_13C_ = 223 ppm)^55^. This chemical shift *δ*_13C_ value is commonly utilized to evaluate the strength of acid sites in solid acids, e.g., a larger *δ*_13C_ value indicates a higher acid strength^6^. Hence, the BAS of SA/10 exhibits a moderate acidity. These sites have been described as SiOH groups in the proximity of one Al^IV^ or Al^V^ site^16^.

### Local structure of SA/50

**Fig. 2:**
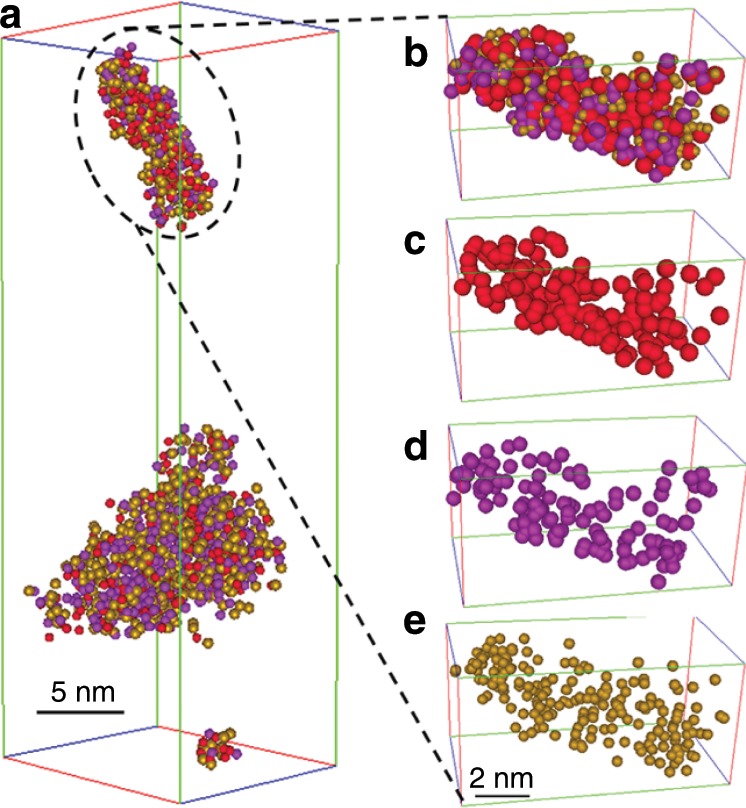
3D-APT reconstruction of two isolated SA/50 nanoparticles. **a** All atoms. **b** Enlargement of the top SA/50 nanoparticle in **a**, showing only **c** Al, **d** Si and **e** O. The 3D-APT reconstruction of the bottom nanoparticle of **a** is shown in Supplementary Fig. 3.

The APT reconstructions of SA/50 in Fig. 2, Supplementary Fig. 3, and Supplementary Movie 2 show the 3D distributions of Al, Si and O species. Visually, the Si and O species are homogeneously distributed, similarly to those observed in Supplementary Fig. 1 for SA/10, however, the Al atoms are distributed rather heterogeneously. The comparison of these APT reconstructions with those in SA/10 (Supplementary Fig. 1) shows a higher Al density in SA/50. Additionally, a radial distribution function (RDF)^56,57^ was calculated to further evaluate the clustering tendency of Al (Supplementary Note 1). The RDF of Al in SA/10 shows no significant positive or negative correlation, which is in a good agreement with the random Al distribution in the silica network without significant clustering in SA/10. The RDF of Al in SA/50 has a high positive correlation indicating that more and more Al species are close to each other. Indeed, at high Al concentration, more Al species in the network lead to shorter average Al-Al distance in SA/50 than in SA/10. Hence, more than one Al center can be expected in the proximity of a SiOH group in SA/50.

**Fig. 3:**
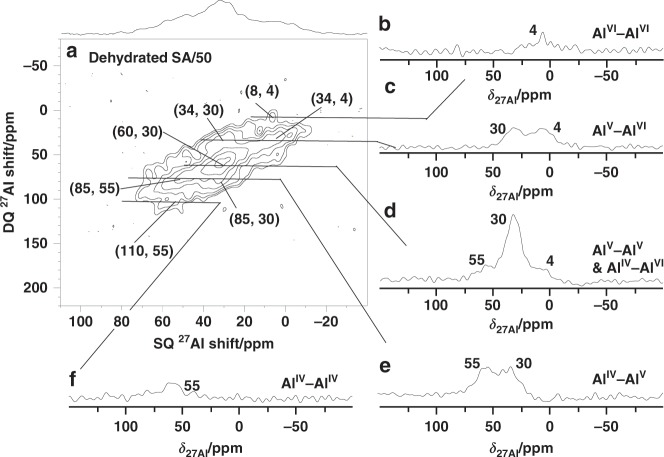
^27^Al DQ-SQ 2D NMR spectrum. **a** ^27^Al 2D NMR spectrum recorded at 18.8 T with *ν*_R_ = 20 kHz of SA/50 dehydrated at 723 K for 12 h under vacuum. **b**–**f** Rows extracted from the 2D spectrum corresponding to the various auto-correlations and cross peaks. All rows are plotted with the same intensity scale.

The ^27^Al 1D NMR spectrum of SA/50 (Supplementary Fig. 4a) indicates the presence of Al^IV^, Al^V^ and Al^VI^ species in that sample. Moreover, the relative amount of Al^V^ sites is higher in SA/50 than in SA/10 (compare Supplementary Figs. 2a and 4a). Various correlation peaks were detected in the ^27^Al DQ-SQ 2D spectrum of dehydrated SA/50 as shown in Fig. 3a, providing information about the proximities between the different Al sites. The most intense peak is the diagonal one of Al^V^ site at (60, 30) ppm (Fig. 3d). When normalized by the number of transients and the Al molar fraction, the intensity of that peak is 3-fold higher for SA/50 than for SA/10 in the spectrum of Fig. 1a. This higher intensity indicates a shorter average distance between the closest Al^V^ sites in SA/50 than in SA/10, in line with the APT data (Supplementary Note 1). The pair of intense cross-peaks at (85, 55) and (85, 30) ppm also indicates that a significant amount of Al^V^ species (*δ*_27Al_ = 30 ppm) is close to Al^IV^ ones (*δ*_27Al_ = 55 ppm) (Fig. 3e). An Al^V^-Al^VI^ correlation is also detected at (34, 4) and (34, 30) ppm (Fig. 3c). The weak cross-peaks at (59, 55) and (59, 4) ppm detected in Fig. 3d also indicate the proximity between Al^IV^ and Al^VI^ species. The weak diagonal signals at (8, 4) or (110, 55) ppm point to proximities between two Al^VI^ or two Al^IV^ sites, respectively. However, owing to the low density of Al^VI^ and Al^IV^ species, these peaks are very weak, as shown in the corresponding slices of Fig. 3b, f, respectively.

The ^1^H 1D NMR spectrum of dehydrated SA/50 (Supplementary Fig. 4b) is dominated by the signal of SiOH groups resonating at 1.9 ppm. The shoulder at 1.1 ppm is assigned to non-acidic terminal Al^VI^OH protons^16^. The ^1^H DQ-SQ spectrum in Supplementary Note 2 exhibits an intense autocorrelation peak at (3.8, 1.9) ppm, indicating close proximity between silanol protons. As mentioned above, the RDF data in Supplementary Note 1 indicates that almost all network Si atoms are close to Al ones. Furthermore, it has been shown for SA/50 using ^1^H-^27^Al through-space correlation experiments at 18.8 T that the silanol protons are mostly close to Al^IV^ and Al^V^ sites^16^. Hence, the intense autocorrelation peak for silanol protons in the ^1^H DQ-SQ 2D spectrum is consistent with the intense Al^IV^-Al^V^ and Al^V^-Al^V^ correlations detected in the ^27^Al DQ-SQ 2D spectrum (Fig. 3d, e).

As already observed for SA/10, the loading of SA/50 with ammonia leads to the appearance of a signal of ammonium protons at 6.7 ppm in the ^1^H 1D NMR spectrum. The relative intensity of this signal with respect to that of silanol is higher for SA/50 than for SA/10, which indicates the increased amount of BAS in SA/50 in agreement with previous studies^5^. The ^1^H DQ-SQ 2D spectrum of SA/50 loaded with ammonia is shown in Supplementary Note 2. The significant increase of the broad auto-correlation signal at (2.6, 5.2) ppm with increasing Al/Si ratio from 1/9 to 5/5 (compare Fig. 1d and Supplementary Note 2) allows assigning it to ammonia adsorbed on LAS^51–54^. Such assignment is supported by previously reported 2D ^27^Al-^1^H through-space correlations of SA/10 and SA/50 loaded with ammonia^16^. In those spectra, the ^1^H signal resonating at 2.6 ppm is mainly correlated with Al^V^ sites, acting as LAS. After ammonia adsorption, the ^27^Al DQ-SQ 2D spectrum is broadened (Supplementary Fig. 5). However, the majority of the Al^IV^-Al^V^ and Al^V^-Al^V^ correlations are still observed. In the ^1^H DQ-SQ spectrum of dehydrated SA50 (Supplementary Note 2) no signal of Al(OH)Al groups at ca. 1.7–2.7 ppm could be detected^6^, indicating a low probability of Al-Al correlations originating from Al^IV^-OH-Al^V^ and Al^V^-OH-Al^V^ groups in alumina domains. Furthermore, these sites do not protonate ammonia and hence, the corresponding ^27^Al correlation will not be significantly broadened in the presence of ammonia. Conversely ammonia can be protonated by surface BAS (SiOH with nearby Al) on ASAs, which explains the peak broadening observed in the ^27^Al DQ-SQ 2D spectrum after ammonia adsorption (Supplementary Fig. 5). This broadening has been proposed by the synergy of Al^IV^-Al^V^ and Al^V^-Al^V^ spin pairs in the local structure of the same SiOH group (Al-SiOH-Al) with enhanced acid strength.

### Acidity enhancement by the synergy of nearby Al species

**Fig. 4:**
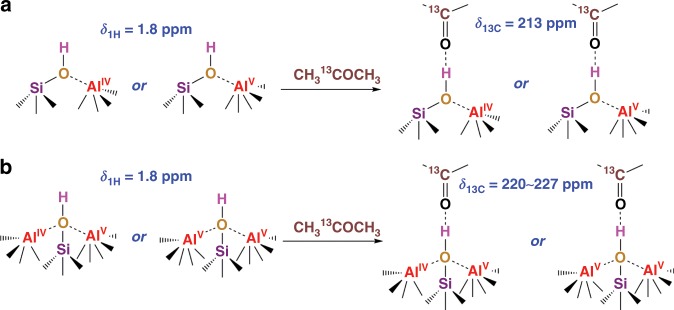
Proposed models for BAS on ASA generated by. **a** one Al center per SiOH for moderate BAS, and **b** two Al centers per SiOH group, leading to moderate or zeolitic acidity strengths. The acid strength is estimated by measuring the ^13^C isotropic chemical shift of CH_3_^13^COCH_3_, which is loaded on dehydrated samples on a vacuum line, followed by evacuation at room temperature for 1 h to remove weakly adsorbed molecules.

In SA/10, as in most ASAs, only a moderate acidity strength (*δ*_13C_ = 213 ppm probed with CH_3_^13^COCH_3_) was detected^5^. Figure 1 and Supplementary Fig. 1 show that the Al^IV^ and Al^V^ species are well-distributed on ASA without obvious correlations, e.g. SA/10. Moreover, bridging OH groups (*δ*_1H_ = 3.6–5.2 ppm) were not detected in ^1^H NMR 1D experiments (Supplementary Fig. 2b). Therefore, the moderate BAS strength in SA/10 is proposed to be generated by one Al center interacting with the neighboring silanol group and in that way decreasing the electron density of the O atom, leading to the formation of one acid SiOH site (BAS), as shown in Fig. 4a. This arrangement is similar to the PBS model proposed in previous theoretical calculation studies, where a SiOH group electrostatically interacts with an acceptor Al center (Al^IV^ or Al^V^), but is not covalently bonded as bridging OH groups in zeolites^24,31^.

In SA/50, two Al centers (two Al^V^ or one Al^V^ and one Al^IV^) can be in the proximity of the same SiOH group. In a previous study, based on DNP (dynamic nuclear polarization) and first-principle calculations, several possible models for two or more Al centers in the vicinity of a SiOH group on ASA, prepared by chemical liquid deposition of SiO_2_ on Al_2_O_3_, have been proposed by Valla and coworkers^21^. In ASAs, BAS are formed at the surface consisting of mixed alumina and silica and thus a wide distribution of Al species in the local structure of Si species can be expected, particularly in the Al-rich phase, as exemplified for ASAs prepared by chemical liquid deposition of SiO_2_ on Al_2_O_3_^21^. However, these models do not account for the enhanced Brønsted acidity of flame-made ASAs with high Al content, such as SA/50. Therefore, here we propose a structural model of BAS, in which Al^IV^ and Al^V^ sites or two Al^V^ sites interact with the same SiOH group (see Fig. 4b), most likely via pseudo-bridging OH groups as often proposed^24,32^, and withdraw electrons from the oxygen of the near SiOH group, thus enhancing its Brønsted acidity. The structural models shown in Fig. 4 bear similarities with oxygen tri- and tetra-coordinated clusters, which have been proposed earlier in ASA prepared by chemical liquid deposition of SiO_2_ on Al_2_O_3_ and aluminosilicates glasses^21,58^. The formation of oxygen tri- or tetra- coordinated clusters is driven by the increased ionicity of these materials at higher Al content due to the difference in electronegativity between Si and Al atoms^31,59^.

However, one or more Al centers solely covalently bound to the Si atoms of the silanols are unable to protonate adsorbed ammonia, since the silanolate cannot be efficiently stabilized after deprotonation^31,60^. Proton transfer from silanol to the guest molecule can be promoted by the stabilization of the conjugated base (silanolate). The silanolate can be stabilized by a neighboring unsaturated Al center via PBS model^31,60–62^, such as proposed in Fig. 4a, which is often characterized by moderate acidity.

Here, we propose a model as shown in Fig. 4b, for the formation of BAS on SA/50 with an acidity comparable to zeolites. Compared to Fig. 4a for SA/10, the second neighboring unsaturated Al center involved as an acceptor is able to further stabilize the formed silanolate, and thus to promote the proton transfer. Besides, the extra Al center(s) may further withdraw electrons from the O atom from neighboring SiOH that significantly enhances the BAS strength (up to *δ*_13C_ = 227 ppm)^5^, to a higher extent than that of zeolite H-ZSM-5 (*δ*_13C_ = 223 ppm)^55^, similar to that of dealuminated zeolite H-Y (*δ*_13C_ = 228 ppm)^17^. The proposed model is similar to those reported for zeolites where an ionic effect induced by extra-framework Al species can enhance the Brønsted acid strength of bridging OH groups^17^. Therefore, the synergy of two Al^V^ or one Al^IV^ and one Al^V^ centers with nearby SiOH groups is expected to significantly enhance the acid strength of BAS in the ASA.

### In situ ^1^H MAS NMR study on acidity enhancement of BAS

In this work, we show that the proximity between one SiOH group and two Al sites can remarkably enhance the Brønsted acidity in ASAs^5,16^. The activation of the C-H bond in hydrocarbon conversion often requires solid acids containing strong BAS. The activation of the C-H bond in benzene, the simplest aromatic compound, has been extensively studied using H/D exchange experiments, which is of great importance to understand the alkylation processes of aromatic compounds^8–12^. The H/D exchange carried out between C_6_D_6_ and surface BAS (bridging OH on zeolite H-ZSM-5 and acidic SiOH groups on ASA) was confirmed by ^1^H solid-state NMR (Supplementary Fig. 6).

On H-ZSM-5 zeolite, the three ^1^H signals of H-ZSM-5 zeolite at 7.5, 4.0 and 1.8 ppm (see Supplementary Fig. 6a) are assigned to hydrogen atoms bound to aromatic rings, bridging OH (SiOHAl) groups and terminal SiOH groups, respectively. After H/D exchange reaction, the intensity of SiOH groups remained unchanged while that of bridging OH groups decreased with increasing intensity of the aromatic hydrogens. This indicates that the H/D exchange occurred between the benzene-*d*_6_ and the bridging OH groups (e.g. SiOHAl), rather than with the terminal SiOH groups (*δ*_1H_ = 1.8 ppm).

**Fig. 5:**
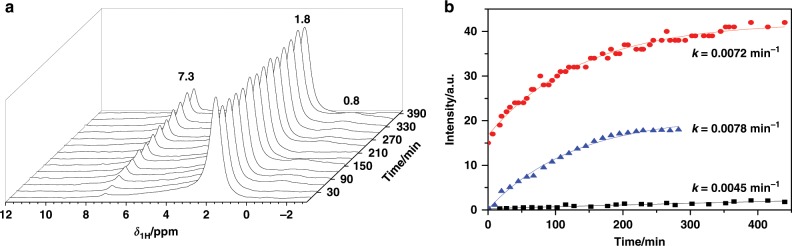
Catalytic performance of ASA in H/D exchange with C_6_D_6_. ^1^H MAS spectra recorded at 9.4 T of dehydrated catalysts, **a** stack plot spectra recorded during H/D exchange of C_6_D_6_ loaded over dehydrated SA/50 at 313 K, with a loading of one benzene molecule per BAS; **b** Kinetics and H/D exchange rates *k* between deuterons bound to the aromatic rings of C_6_D_6_ (99.6 %) and BAS at 313 K in H-ZSM-5 (top), SA/50 (middle) and SA/10 (bottom).

Conversely, no bridging OH groups could be observed at 3.5–5.2 ppm on SA/50 (see Supplementary Fig. 6b)^6^, while the signal of protons bound to the aromatic rings was observed at 7.3 ppm. As shown in the stack plot of the ^1^H MAS spectra recorded during H/D exchange of benzene-*d*_6_ loaded over dehydrated SA/50 (Fig. 5a), the intensity of the terminal SiOH groups (*δ*_1H_ = 1.8 ppm) decreased as a function of time, while that of protons bound to aromatic rings (*δ*_1H_ = 7.3 ppm) increased. This demonstrates that the H/D exchange occurred between C_6_D_6_ and the acidic SiOH groups of ASA. It must be reminded that Lewis acid aluminum sites cannot exchange H with C_6_D_6_ (see Supplementary Note 3).

The reaction rate, *k*, for the C_6_D_6_–SiOH exchange can be utilized to evaluate the relative strength of BAS in silica-alumina catalysts under the same conditions. As described in the Methods section, one molecule of C_6_D_6_ per Brønsted acid site was quantitatively loaded, and thus, a higher *k* value indicates a higher acid strength^63–65^. The rate of the H/D exchange reaction over different catalysts upon loading one C_6_D_6_ per BAS at 313 K was determined by fitting the evolution of the signal intensity of aromatic protons as a function of reaction time^9,12^. The obtained *k* values are shown in Fig. 5b. Evidently, SA/10 with mainly moderate BAS was virtually inactive in the reaction, resulting in a small *k* value, while zeolite H-ZSM-5 with strong BAS provided a much higher reaction rate (*k* = 0.0072 min^−1^). A similar value, *k* of 0.0078 min^−1^, was obtained with SA/50 under the same conditions, hinting to the existence of strong BAS in SA/50 with a strength comparable to that of zeolite H-ZSM-5. Considering the structural difference between SA/10 and SA/50, it shows that the proximity between more than one Al center and SiOH group in ASA with high Al content, such as SA/50, gives rise to BAS with zeolitic strength.

### Stability test of ASA catalysts

Besides their enhanced surface Brønsted acidity, the high stability of ASAs under various conditions is crucial for efficient catalysis. Current ASAs were thus firstly tested in liquid-phase glucose dehydration to 5-hydroxymethylfurfural (HMF), requiring LAS for glucose isomerization to fructose and BAS for fructose dehydration^48^. The catalytic reaction results are summarized in Supplementary Note 4, 5, and Supplementary Fig. 7. The reusability of ASA was tested with SA/50, which exhibited the best performance under the same conditions. After five recycle runs, no significant loss of catalytic activity could be observed (Supplementary Note 5). This is attributed to the high stability of SA/50 in the liquid phase glucose dehydration. In liquid phase reactions under heating, dealumination could cause the modification of surface acid sites. The comparison of Supplementary Fig. 12a, b demonstrates that aluminum species in SA/50 are stable without dealumination upon water treatment at 433 K for 2 h (Supplementary Note 6). Therefore, the synergy of Lewis acidic Al^V^ and enhanced Brønsted acidity renders these ASAs promising bifunctional catalysts for Brønsted-Lewis acid-catalyzed reaction, such as the glucose dehydration to HMF.

It is noteworthy to point out the high stability of the BAS with enhanced strength when they are exposed to high temperature (1073 K) calcination (regeneration temperature in fluid catalytic cracking, Supplementary Fig. 12c) or to a liquid-phase conversion of glyceraldehyde in ethanol (Supplementary Fig. 12d). Two or more Al centers nearby SiOH groups in the amorphous silica network did not leach out from the catalysts after five recycle uses in a batch reaction with ultrasonic washing (Supplementary Fig. 12d), as confirmed by the lack of detectable Al species in the reaction mixture. Conversely, a similar treatment caused strong leaching of extra-framework aluminum species in zeolites, which exhibited enhanced acidity of BAS via the synergy of extra-framework aluminum and BAS. This leaching led to a significant activity loss of these zeolites (Supplementary Note 7) as confirmed by 1D ^27^Al NMR experiments (Supplementary Note 8).

## Discussion

In conclusion, a remarkable synergy between two Al centers (Al^V^-Al^IV^ or Al^V^-Al^V^) close to the same SiOH group has been evidenced in flame-made amorphous silica-alumina (ASA) by 2D ^27^Al and ^1^H DQ-SQ NMR experiments, and analysis of the 3D spatial elemental distribution of Al and Si by APT. The study revealed that compared to the widely accepted model of one Al center per SiOH group with moderate strength (*δ*_13C_ = 213 ppm)^5^, two proximate Al centers can strongly decrease the electron density from a neighboring silanol oxygen and thereby can significantly boost its acid strength (with *δ*_13C_ = 227 ppm for CH_3_^13^COCH_3_) to a value higher than that of H-ZSM-5 (*δ*_13C_ = 223 ppm)^55^, or even reaching that of dealuminated zeolite HY (*δ*_13C_ = 228 ppm)^17^. These BAS with zeolitic strength have been evidenced by comparative H/D exchange experiments with C_6_D_6_. Furthermore, the synergy between BAS with zeolitic strength and LAS afforded a much higher HMF yield (38%) than catalysts with moderate BAS strength (e.g. SA/10 and [Al]MCM-41). The achieved yield was comparable to that realized with metal-doped zeolites (33%) at a higher temperature. The present study highlights a promising route for generating BAS with zeolitic strength and high stability on ASAs, which could facilitate improved catalytic performances in a wide range of applications, including acid and multifunctional catalysis.

## Methods

### APT sample preparation and measurement method

A drop of the diluted dispersed ASA nanoparticles in methanol (≈0.01 mol/L) was placed onto a Si flat wafer, which was covered by a 150-nm thick protective Cr layer in Leica EM ACE600. Needle-shaped APT specimens were prepared from the Si flat sample by a site-specific lift-out procedure using a FEI G4 CX focused ion beam (FIB)/scanning electron microscope^66^. The APT experiments were conducted on a CAMECA LEAP 5000 XR instrument equipped with an ultraviolet laser with a spot size of 2 µm and a wavelength of 355 nm. The detection efficiency of this state-of-the-art microscope is ca. 54%. Data were acquired in laser pulsing mode at a specimen temperature of 50 K, with a target evaporation rate of 3 ions per 1000 pulses, a pulsing rate of 200 kHz, and a laser pulse energy of 50 pJ. The APT data were reconstructed and analyzed using the commercial IVAS 3.6.14™ software.

### NMR experimental details

Before each experiment, the samples in glass tubes were dehydrated at 723 K for 12 h at a pressure lower than 10^−2^ bar. Subsequently, the samples were transferred into the MAS NMR rotors under dry N_2_ inside a glove box. These ammonia-loaded samples were prepared by dehydrated samples loaded with ammonia on a vacuum line, followed by evacuation at 393 K for 1 h to remove weakly physisorbed molecules.

All ^1^H and ^27^Al NMR spectra were recorded on a Bruker Avance III 800 MHz spectrometer equipped with 3.2 mm MAS rotors spinning at 20 kHz. For ^1^H DQ-SQ 2D experiments, the ^1^H DQ coherences were excited and reconverted by applying the symmetry-based R$12_2^5$ scheme^67^, which reintroduces the ^1^H-^1^H dipolar interactions under MAS. The ^1^H radio frequency (rf) amplitudes for the π/2 pulse and R$12_2^5$ scheme were equal to *ν*_1_ = 75 and 60 kHz, respectively. The length of the excitation recoupling scheme was equal to that of the reconversion and ranged from 250 to 300 μs, depending on the experiment. ^1^H DQ-SQ 2D spectra resulted from averaging 32 to 128 transients with recycle delay of 1 to 5 s, resulting in a total experimental time of 2 to 4 h. During ^27^Al DQ-SQ 2D experiments, selective central transition (CT) π/2 and π-pulses of 8 and 16 μs, that is, an rf amplitude of about 10 kHz, were applied. The ^27^Al two-spin DQ coherences were excited and reconverted by applying the BR$2_2^1$ pulse sequence^36^, which reintroduces the ^27^Al−^27^Al dipolar interactions under MAS. The lengths of the excitation and reconversion periods were equal and ranged from 800 to 1200 μs, depending on the experiment. The rf amplitude applied during the BR$2_2^1$ pulse sequence was 6.6 kHz, which corresponds to a nutation frequency of 20 kHz for the ^27^Al CT. Furthermore, the Hyper–Secant (HS) scheme was applied before the BR$2_2^1$ excitation^68^, in order to enhance the ^27^Al CT polarization by saturating the satellite transitions^69,70^. HS employed a shaped pulse lasting 4 ms with an rf field amplitude of 16 kHz and a frequency sweep of 20 kHz around an offset of 200 kHz with respect to the CT. ^27^Al DQ-SQ 2D spectra resulted from averaging 14,400 and 3200 transients for SA/10 (Fig. 1) and SA/50 (Fig. 3) with recycle delay of 0.2 s, resulting in a total experimental time of 25.3 and 5.7 h, respectively. The ^1^H isotropic chemical shifts were referenced to tetramethylsilane using the resonance of adamantane (1.83 ppm) as a secondary reference, whereas the ^27^Al ones were referenced to 1 M solution Al(NO_3_)_3_.

### In situ ^1^H MAS NMR Spectroscopy of H/D exchange with C_6_D_6_

^1^H MAS NMR spectra of H/D exchange with C_6_D_6_ was carried out on a Bruker Avance III 400 WB spectrometer at the Larmor frequency of 400.1 MHz with 4 mm MAS rotors spinning at 8 kHz. Spectra were recorded after single-pulse π/2 excitation with repetition times of 20 s and 8 scans. Prior to measurements, all samples were dehydrated at 723 K in vacuum (pressure <10^−2^ bar) for 12 h in glass tubes. The density of BAS on all dehydrated samples was determined by quantitative ^1^H MAS NMR experiments using NH_3_ as probe molecules. The total number of BAS was calculated based on the BAS density and weight of the sample. A known amount of dehydrated samples was transferred into the MAS rotors under dry nitrogen gas inside a glove box, sealed and utilized for in-situ loading on a vacuum line. The loading pressure of benzene-*d*_6_ (99.6%, Cambridge Isotope Laboratories, Inc.) was calculated and controlled according to the total number of BAS and known volume of the vacuum line to ensure one molecule of benzene-*d*_6_ per BAS. Then the sample was cooled down by liquid nitrogen till nearly no pressure could be detected. Subsequently, the loaded samples in the MAS rotors were kept 10 min at room temperature under dry nitrogen gas inside a glove box for better diffusion. The H/D experiments were performed by heating the MAS rotor at 313 K in a variable-temperature probe for ^1^H MAS NMR investigations. The concentration of protons bound to the aromatic rings was calculated as the ratio between the integrated intensity of the aromatic ^1^H signal and the number of Brønsted acid sites. The rate *k* of the H/D exchange between the deuterated molecules and the acidic OH groups (BAS) is described by an exponential relationship^9^

1 $$I\left( t \right) = I\left( \infty \right)\left[ {1 - b{\mathrm{exp}}\left\{ { - kt} \right\}} \right]$$

where *I*(*t*) and *I*($\infty$) denote the intensities of the ^1^H MAS NMR signal of the aromatic rings at the observation time *t* and *t* → + $\infty$ in the equilibrium state, respectively. The *b* parameter describes the exchange at *t* = 0, which corresponds to the start of the H/D exchange experiment, i.e. when the temperature was increased from ca. 293 K to that of the reaction.

## Supplementary information

Supplementary Information

Description of Additional Supplementary Files

Supplementary Movie 1

Supplementary Movie 2

## Data Availability

Raw data are available from the authors upon reasonable request.
