# Supplementary material for: Acidity enhancement through synergy of penta- and tetra-coordinated aluminum species in amorphous silica networks
Source: Nat Commun. 2020 Jan 13;11:225. doi: 10.1038/s41467-019-13907-7 (PMC6957685; doi:10.1038/s41467-019-13907-7)
Supplement: Supplementary file 1 — Supplementary Information [file 41467_2019_13907_MOESM1_ESM.pdf]

Supplementary Information

Acidity enhancement through synergy of penta- and tetra-coordinated aluminum species in amorphous silica networks

Wang. et al.

Supplementary Methods

Amorphous silica-alumina sample preparation method

Amorphous silica-alumina (ASA) catalysts were prepared by flame-spray pyrolysis (FSP) within microseconds, which could combine both synthesis and calcination in a single step at extremely high temperature (ca. 2000 K). Briefly, the appropriate amount of the precursor materials were dissolved in a 1:1 (vol. %) mixture of acetic acid and methanol. The resulting solution was filtered using a glass filter, pumped through a capillary at a rate of 5 $mLmin^{-1}$, and nebulized at 5 $Lmin^{-1}$ O_2, and finally ignited by an annular supporting methane/oxygen flame (1.5/0.9 $Lmin^{-1}$) to generate ASA nanoparticles. The silica-alumina catalysts are designated as SA/x, where $x = n_{Al}/(n_{Al} + n_{Si}) \times 100$, indicates mol% of the Al precursor in the precursors.

NMR experimental details for 1D NMR measurements

Before each experiment, the samples in glass tubes were dehydrated at 723 K for 12 h at a pressure lower than 10^{-2} bar. Subsequently, the samples were transferred into the MAS NMR rotors under dry N_2 inside a glove box. These ammonia-loaded samples were prepared by dehydrated samples loaded with ammonia on a vacuum line, followed by evacuation at 393 K for 1 h to remove weakly physisorbed molecules.

All ^{1}H and ^{27}Al NMR spectra were recorded on a Bruker Avance III 800 MHz spectrometer equipped with 3.2 mm MAS rotors spinning at 20 kHz. The ^{1}H spectra (except those of Supplementary Fig. 11) were acquired using the DEPTH sequence[1] in order to suppress the ^{1}H background signal due to the rotor and the probe. For DEPTH experiments, the rf nutation frequency of the pulses was 75 kHz. The ^{1}H DEPTH 1D spectra result from the averaging of 16 transients with a recycle delay of 5 s. The ^{27}Al 1D

NMR spectra were acquired using a pulse of 0.9 μs and an rf field of ν_1 = 100 kHz. The ^{27}Al 1D NMR spectra resulted from the averaging of 2048 transients with recycle delay of 0.4 s for SA/10 (Supplementary Fig. 2), 512 transients with recycle delay of 4 s for SA/50 (Supplementary Fig. 4). The ^{1}H isotropic chemical shifts were referenced to tetramethylsilane using the resonance of adamantane (1.83 ppm) as a secondary reference, whereas the ^{27}Al ones were referenced to 1 M solution $Al(NO_3)_3$.

^{27}Al and ^{13}C MAS 1D spectra of Supplementary Figs. 7,10-12 and 15 were acquired on a Bruker Avance III 400 WB spectrometer at Larmor frequencies of 104.3, and 100.6 MHz, respectively, and with a 4 mm MAS rotor spinning at 8 kHz. The spectra were recorded after a single-pulse π/6 excitation with repetition times of 0.5 s and 4,800 scans. $^{1}H \rightarrow ^{13}C$ cross-polarization (CP) MAS NMR spectra were recorded with a contact time of 4 ms and a repetition time of 4 s, after 20,000 number of scans.

Glucose dehydration to 5-Hydroxymethylfurfural (HMF)

Prior to the reaction, the catalyst ([Al]MCM-41 and ASA) was pre-heated overnight at 723 K under a nitrogen flow of 50 mL/min. A certain amount of glucose (60 mg) was dissolved in a mixture of deionized water (0.6 mL) and DMSO (1.4 mL) as organic phase. Then, the mixture was transferred into a glass pressure reactor (25 mL) together with the pre-heated catalyst (0.02 g). The reaction was performed at 433 K under magnetic stirring for 4 h in an oil bath. Samples of the reaction mixture were collected after 15, 30, 45, 60, 90, 120, 180, 240 min, diluted by 20.0% (v/v) methanol aqueous solution and filtered for HPLC analysis. HMF was quantified using a reversed-phase C18 column and a multi-wavelength detector at 284 nm connected to an Agilent HPLC. The mobile phase was 20.0% (v/v) methanol aqueous solution at a flow rate of 0.6 mL/min.

Supplementary Figures

a) b) c) d)

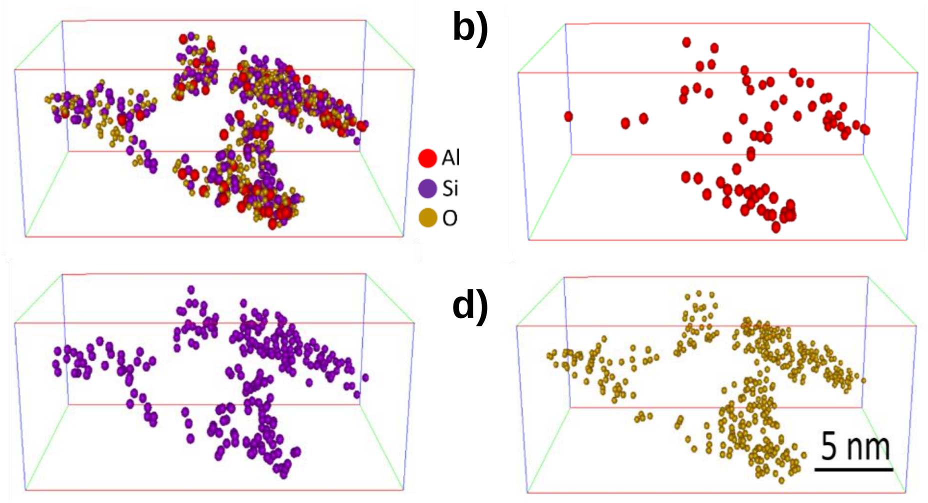

Supplementary Figure 1. APT detection and homogeneous distribution of aluminum atoms in reconstructed 3D atom distribution maps of SA/10 catalyst nanoparticles: (a) all atoms, (b) Al, (c) Si, and (d) O. Bounding box dimensions are 35 nm (length) × 8 nm (width) × 12 nm (depth). Note that the detection of H atoms by APT is challenging due to the existence of residual hydrogens in the ultra-high vacuum chamber.

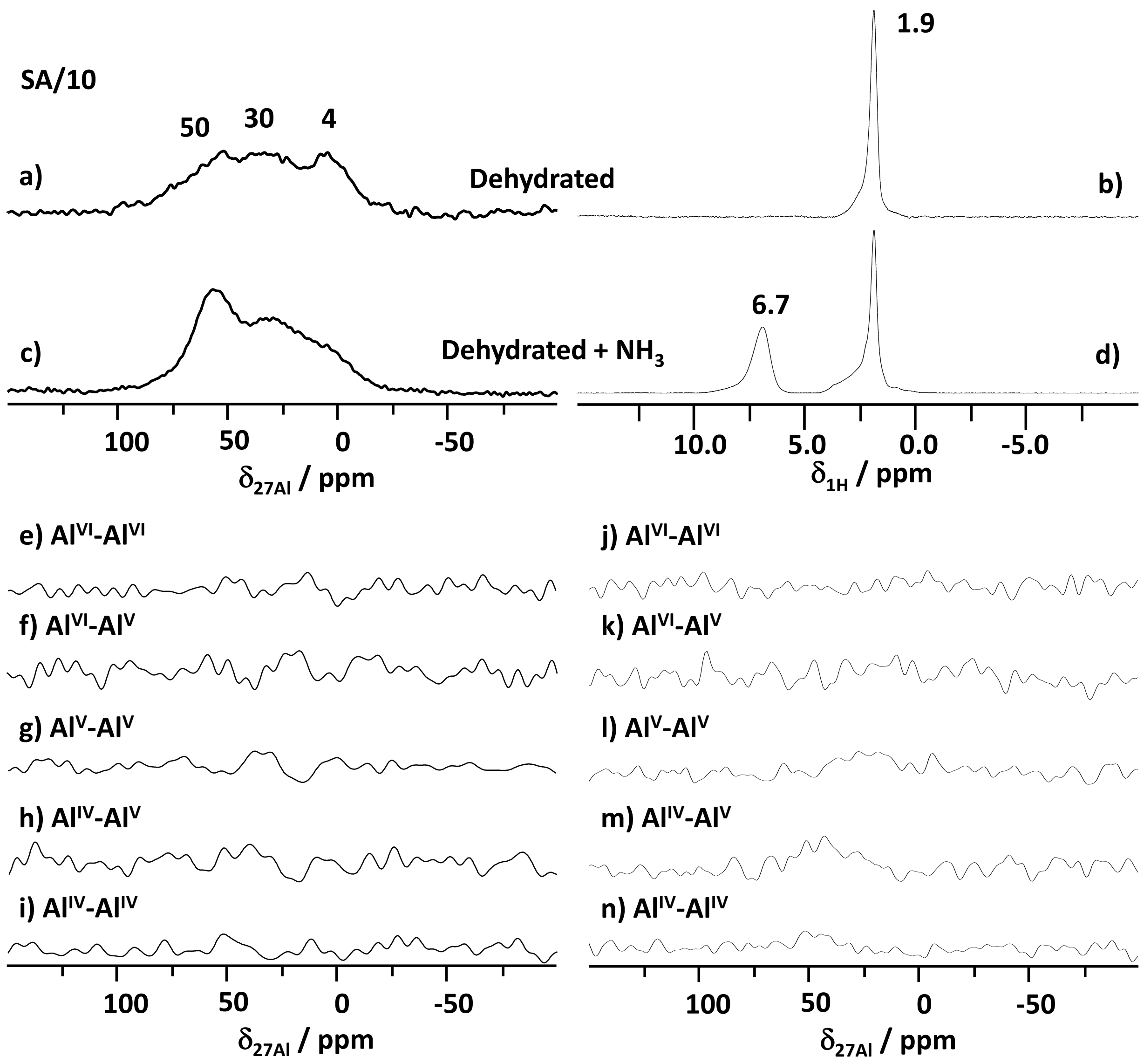

Supplementary Figure 2. ^{27}Al (a, c) and ^{1}H (b, d) MAS NMR spectra of SA/10: (a, b) dehydrated at 723 K for 12 h under vacuum and (c, d) followed by ammonia loading and evacuation at 393 K for 1 h to remove the weakly physisorbed molecules. ^{27}Al slices at the shifts of Al^{VI}-Al^{VI}, Al^{VI}-Al^{V}, Al^{V}-Al^{V}, Al^{IV}-Al^{V},and Al^{IV}-Al^{IV} signals of dehydrated SA/10 (e-i) and of ammonia-loaded SA/10 (j-n), extracted from the 2D spectrum in Fig. 1a and c, respectively, referred to Fig. 3a. Spectra were recorded at 18.8 T with ν_R = 20 kHz.

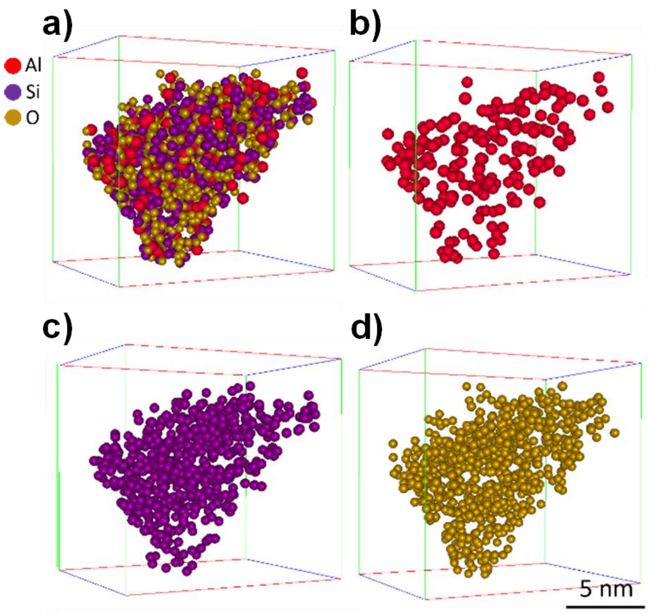

Supplementary Figure 3. APT detection and homogeneous distribution of aluminum atoms in reconstructed 3D elemental distribution maps of SA/50 catalyst nanoparticles showing: (a) all atoms, (b) Al, (c) Si, and (d) O. Bounding box dimensions are 35 (length) × 8 (width) × 12 (depth) nm^3. Note that the detection of H atoms by APT is challenging due to the existence of residual hydrogens in the ultra-high vacuum chamber.

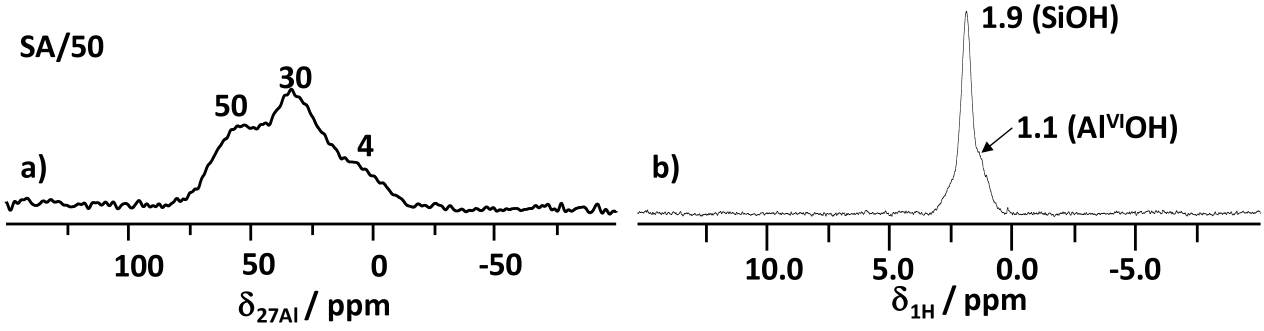

Supplementary Figure 4. ^{27}Al (a) and ^{1}H (b) MAS NMR spectra of SA/50: dehydrated at 723 K for 12 h under vacuum. Spectra were recorded at 18.8 T with ν_R = 20 kHz.

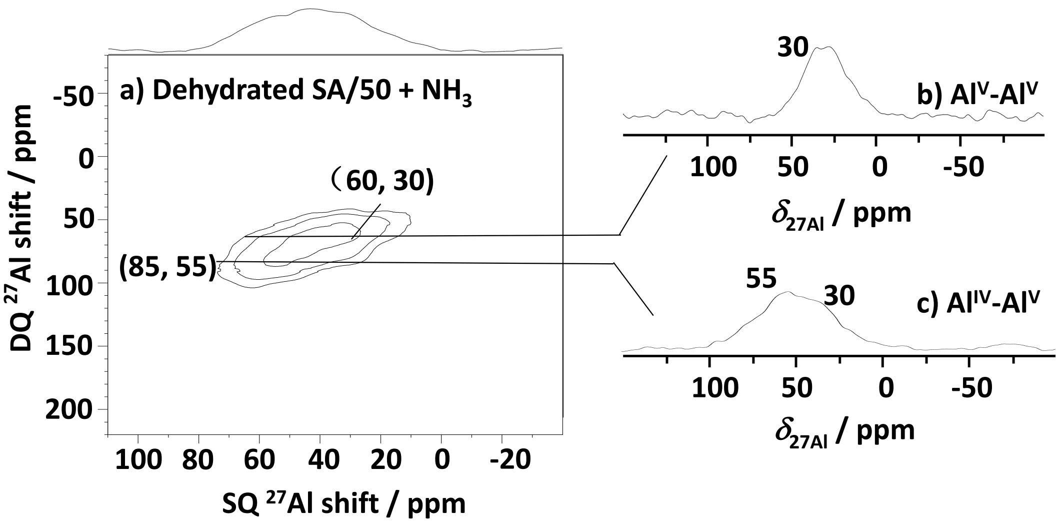

Supplementary Figure 5. (a) ^{27}Al DQ-SQ 2D NMR spectrum recorded at 18.8 T with ν_R = 20 kHz of dehydrated SA/50 upon ammonia loading and evacuated at 393 K for 1 h to remove the weakly physisorbed molecules. (b,c) rows of 2D spectrum corresponding to the (b) Al^{V}-Al^{V} and (c) Al^{IV}-Al^{V} correlations.

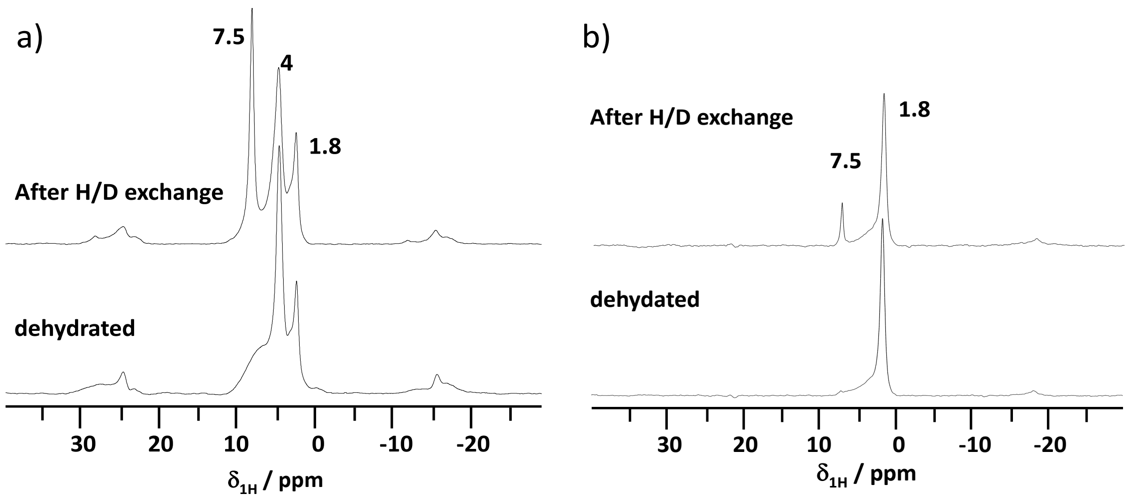

Supplementary Figure 6. Catalytic performance of ASA in H/D exchange with d_6-benzene. ^{1}H MAS NMR spectra at 9.4 T of dehydrated catalysts, (a) H-ZSM-5 and (b) SA/50, before and after H/D exchange with benzene-d_6 at 313 K with a loading of one benzene per BAS.

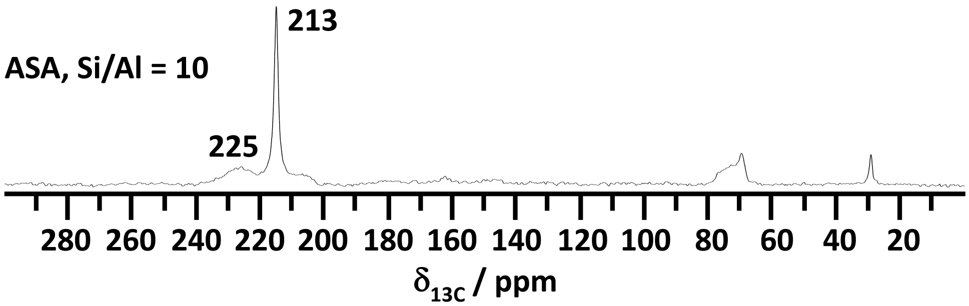

Supplementary Figure 7. $^{1}H \rightarrow ^{13}C$ CP/MAS spectrum of conventional ASA prepared by co-precipitation method[2] with Si/Al = 10, recorded after ASA was dehydrated at 723 K for 12 h, then *in situ* loaded with [^{13}C]-2-acetone (99.5% ^{13}C-enriched, Sigma–Aldrich) on a vacuum line, followed by evacuation at room temperature for 10 min. The signals at 213 and 225 ppm indicate the presence of BAS and LAS on ASA, respectively. Compared to flame-derived SA/10[3], the LAS density on this reference ASA is much higher.

Supplementary Notes

Supplementary Note 1. APT data analysis

In order to evaluate the clustering tendency of Al, the radial distribution function (RDF)[4-5] was calculated at every Al ion center in the APT data of SA/50 and SA/10 shown in Fig. 2 and Supplementary Fig. 1. For a random distribution, the bulk normalized concentration is 1. If it is more than 1, it suggests a positive correlation or a clustering tendency for the solute. If it is less than 1, it indicates a negative correlation. The RDF of Al in SA/10 shows no significant positive or negative correlation, while that in SA/50 has a high positive correction (Supplementary Fig. 8).

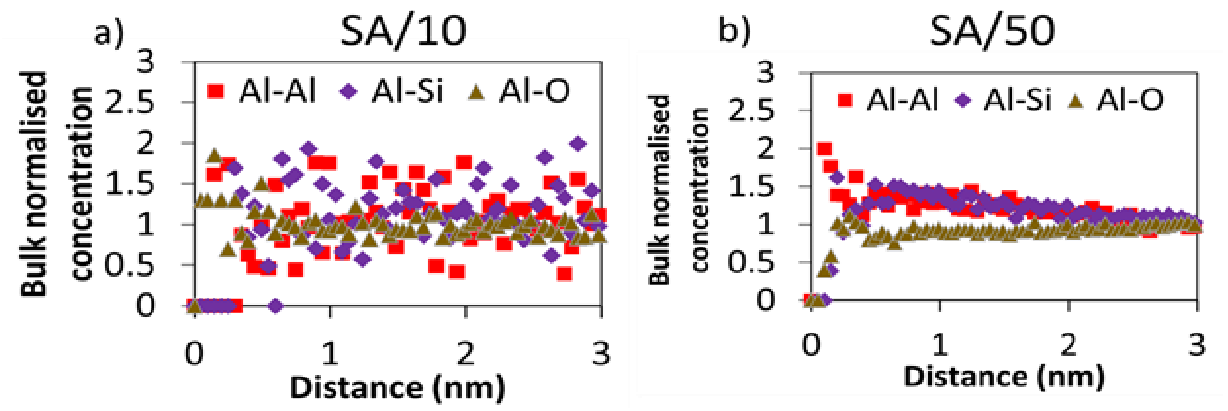

Supplementary Figure 8. Radial distribution function from Al centers in APT data of a) SA/10 and b) SA/50.

Supplementary Note 2. ^{1}H DQ-SQ *D*-HOMCOR 2D spectra of SA/50

The ^{1}H DQ-SQ spectrum of Supplementary Fig. 9a displays only autocorrelation peaks at (2.2, 1.1) and (3.8, 1.9) ppm, which indicate neighboring Al^{VI}OH and silanol groups, respectively. Therefore, there is a close proximity in SA/50 samples between ^{1}H environments of the same type, whereas no proximity between Al^{VI}OH and SiOH protons is detected. The most intense correlations observed in the ^{1}H DQ-SQ spectrum of SA/50 loaded with ammonia shown in Supplementary Fign 9b are: (i) the auto-correlation peaks of silanol protons at (3.8, 1.9) ppm, ammonia adsorbed on LAS at (5.2, 2.6) ppm and ammonium ions at (13.4, 6.7) ppm, as well as (ii) the cross peaks at (8.6, 6.7) and (8.6, 1.9) ppm between ammonium ions and silanol protons. These cross-peaks indicating a close proximity between ammonium ions and silanol groups are consistent with the close proximity between silanol sites, which is deduced from the 2D spectrum of Supplementary Fig. 9a. The ammonia, which reacts with a BAS, forms an ammonium ion, which remains close to the neighboring silanol groups.

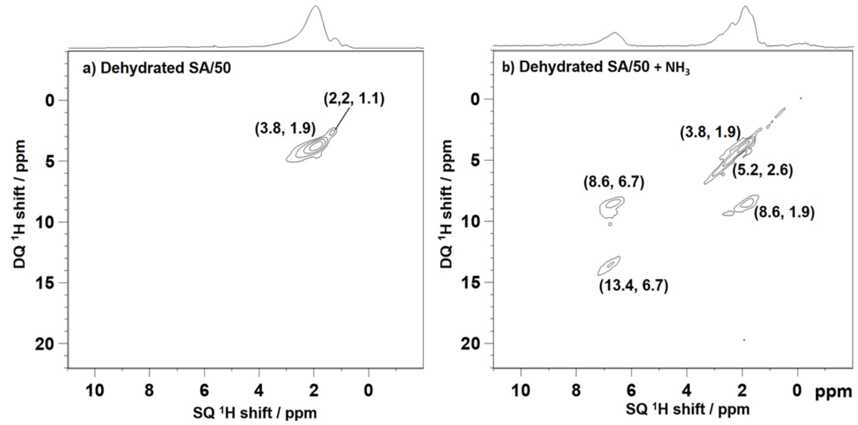

Supplementary Figure 9. ^{1}H DQ-SQ spectra of SA/50: (a) dehydrated at 723 K for 12 h under vacuum and (b) followed by ammonia loading and evacuation at 393 K for 1 h to remove the weakly physisorbed molecules. Spectra were recorded at 18.8 T with ν_R = 20 kHz.

Supplementary Note 3. H/D exchange of *d*$_6$-benzene on Al_2O_3

Lewis acid aluminum sites have no H and are therefore unable to exchange H with C_6D_6. Only hydrated alumina or undehydrated alumina may possibly contain active OH groups for H/D exchange. This has been evidenced by performing H/D exchange with dehydrated Al_2O_3 under the same conditions. As shown in the Supplementary Fig. 10, no reaction can be observed even after 90 min when loading C_6D_6 at a higher temperature (353 K) than that used for ASA (313 K).

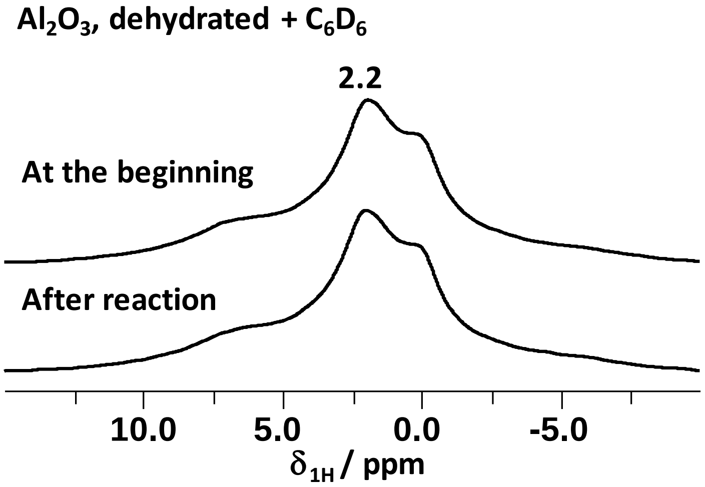

Supplementary Figure 10. Catalytic performance of Al_2O_3 in H/D exchange with *d*$_6$-benzene. ^{1}H MAS NMR spectra at 9.4 T of dehydrated catalysts, recorded during H/D exchange of benzene-*d*$_6$ loaded over dehydrated Al_2O_3 at 353 K.

Supplementary Note 4. ^{27}Al 1D spectrum of [Al]MCM-41, Si/Al = 10

As shown in Supplementary Fig. 11, the strong peak at 54 ppm is attributed to framework Al^{IV} species, contributing to the formation of surface BAS on [Al]MCM-41. The peak at ca. 0 ppm, assigned to Al^{VI} species, indicates the presence of non-framework Al species on [Al]MCM-41, which often act as surface LAS in reactions.

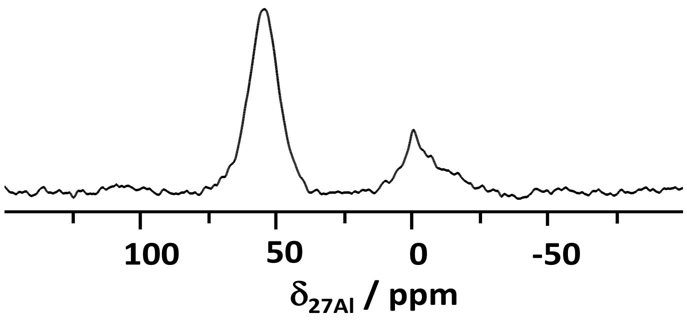

Supplementary Figure 11. ^{27}Al single-pulse MAS spectrum of [Al]MCM-41 recorded at 9.4 T with ν_R = 8 kHz.

Supplementary Note 5. Glucose dehydration to HMF over ASA catalysts.

Besides enhancing the surface Brønsted acidity on ASA, Al^{V} species are widely accepted as surface LAS. Al^{V}-enriched ASAs (e.g. SA/50) are thus potential bifunctional Brønsted-Lewis acid catalysts in the conversion of glucose to HMF, requiring LAS for glucose isomerization to fructose and BAS for fructose dehydration.[6-7] Therefore, zeolite H-ZSM-5 with exclusive BAS is inactive in the production of HMF as reported in the literature.[8] As shown in Supplementary Table 1, introducing Lewis acidity on H-ZSM-5 zeolites through ion-exchange with Fe(II) precursor (Fe-ZSM-5) can significantly increase the HMF yield to 33 % at a conversion of ca. 90 %. [Al]MCM-41, with larger pore sizes and much lower densities of BAS and LAS than zeolites, can provide a similar HMF yield of 36 % at 87 % glucose conversion under the same conditions, which is significantly reduced to only 4 % HMF yield at lower reaction temperatures (e.g. 438 K).[9] Under similar conditions, a comparable HMF yield of 3 % was obtained in this work using [Al]MCM-41 (Si/Al = 10, see ^{27}Al NMR spectrum in Supplementary Fig. 10).

The catalytic performance of ASA was examined in the conversion of glucose to HMF and the results are summarized in Supplementary Table 1. Flame-derived SA/10 afforded a HMF yield of 6.3 %, significantly lower than that of 18.8 % obtained with conventional ASA (Si/Al = 10) prepared by co-precipitation.[2] The lack of LAS on SA/10 compared to conventional ASA, has been evidenced by $^{1}H \rightarrow ^{13}C$ NMR spectra (ref.[6] and Supplementary Fig. 11). This absence may hamper the isomerization of glucose (conversion 23 vs. 45 %), leading to a lower HMF yield. Introducing a high content of surface Al^{V} species (Supplementary Figs. 2a vs. 5a), as in SA/50, provided a much higher HMF yield of 38 %, which is even higher than that obtained with Fe-ZSM-5 at higher

reaction temperature (468 vs. 433 K) and longer reaction time (2.5 vs. 2 h). Al^{V} and Al^{VI} are associated to the formation of Lewis acidity on ASA. In SA/50, Al^{V} is the dominant Al species while only a small amount of Al^{VI} (< 3 %) can be detected.[10] It has been demonstrated that the Al^{V} centers can contribute to enhancing the Brønsted acidity on ASA. Moreover, the numerous Al^{V} centers on the surface can also promote the Lewis acidity of SA/50, as indicated by the cross peak between NH_3 protons resonating at 2.6 ppm and the Al^{V} sites in the $^{27}Al\{^{1}H\}$ *D*-HMQC 2D spectrum of SA/50.[10-11] This cross peak proves the adsorption of NH_3 on the surface of Al^{V}-based LAS. The high HMF yield can be attributed to the high content of Al^{V} species and their close spatial proximity on SA/50 (Fig.**3**), which can i) enhance the surface Lewis acidity to improve the glucose isomerization; and ii) boost the Brønsted acidity for the efficient dehydration of fructose to HMF.

Supplementary Table 1. Catalytic performance of ASA and pertinent reference catalysts in the conversion of glucose to HMF.[a]

	Temp (K)	Time (h)	Conversion (%)	Yield (%)	Ref.
Fe-ZSM-5, Si/Al = 22.8 [b]	468	2.5	90	33	8
[Al]MCM-41, Si/Al = 10 [b]	438 (468)	2.5	32 (87)	4 (36)[b]	9
[Al]MCM-41, Si/Al = 10	433	2	30	3	This work
ASA[c], Si/Al = 90/10	433	2	45	18.8	
SA/10, Si/Al = 90/10	433	2	23	6.3	
SA/50, Si/Al = 50/50	433	2	70 (68)	38 (37)[d]	

[a] Conditions: catalyst (0.02 g) was added to a mixture of deionized water (0.6 mL) and DMSO (1.4 mL) containing glucose (0.06 g). [b] Conditions: catalyst (0.05 g) was added to a mixture of deionized water (1.5 mL) and MIBK (3.5 mL) containing glucose (0.15 g). Fe/Al = 0.21. HMF yields obtained at 468 K are given in parentheses. [c] Conventional ASA prepared by co-precipitation. [d] The catalytic data after five recycle runs are given in parentheses.

Supplementary Note 6. ^{27}Al 1D spectra of SA/50 and reaction mixture

The comparison of Supplementary Fig.s 12a and 12b demonstrates that aluminum species in SA/50 are highly stable showing no dealumination process upon water treatment under 433 K for 2 h in liquid phase glucose dehydration, which is confirmed by the lack of Al signals detected by ICP-AES analyses of the reaction mixture. The aluminum species in SA/50 were also stable after calcination at 1073 K for 2 h (Supplementary Fig. 12c), which is a standard regeneration temperature in fluid catalytic cracking. Furthermore, SA/50 has been employed in the catalytic conversion of glyceraldehyde in ethanol to yield ethyl lactate. After five recycle uses, no significant change could be detected in the 27Al spectra of SA/50 (Supplementary Fig. 12d) and no Al species could be detected in the reaction mixture. This indicates that the AlV species are highly stable in both gas- and liquid-phase reactions. As a reference, dealuminated zeolite was applied in the same reaction. However, leaching of extra-framework aluminum species has been observed, leading to significantly activity loss and recycle use issues, as shown in the Supplementary Fig. 14.

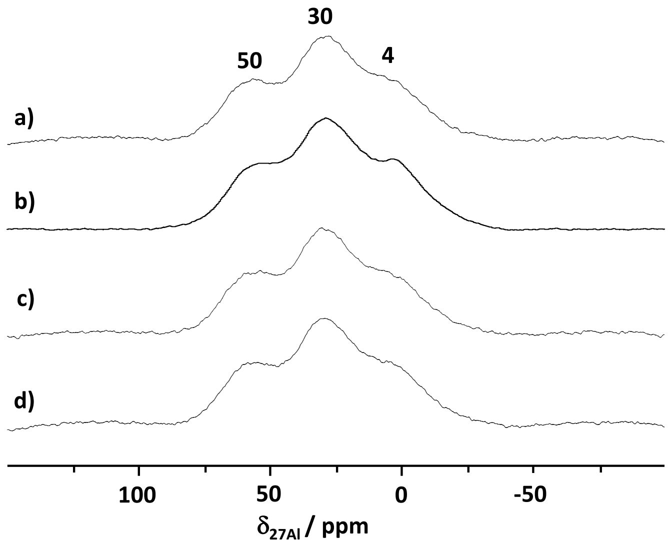

Supplementary Figure 12. ^{27}Al single-pulse MAS spectra recorded at 9.4 T with ν_R = 8 kHz for SA/50: (a) dehydrated at 723 K for 12 h under vacuum; (b) after glucose dehydration at 433 K for 2 h, followed by calcination at 773 K in air to remove organics and dehydration as sample (a); (c) calcination at 1073 K (a typical regeneration temperature in fluid catalytic cracking) for 2 h; (d) after five recycle uses (recycle method as described earlier[12]) in the catalytic conversion of glyceraldehyde in ethanol, followed by calcination at 773 K in air to remove organics and dehydration as sample (a).

Supplementary Note 7. Catalytic conversion of glyceraldehyde to ethyl lactate

As shown in Supplementary Fig. 13, the conversion of glyceraldehyde increased as a function of time over all catalysts. Dealuminated HY zeolite (De-Al-HY) exhibited a much higher activity than the parent HY zeolite, which is attributed to the presence of extra-framework Al in De-Al-HY.[13] Over ASA catalysts, the enhanced acidity at higher Al content can strongly promote the conversion of glyceraldehyde. For comparison, SA/50 provides a higher activity than De-Al-HY zeolite in the first 5 h of reaction time. However, a sharp increase of glyceraldehyde from 49% to 93% was observed with De-Al-HY zeolite after 5 h of reaction time. Such increase has been explained by the extra-framework Al species leaching out from De-Al-HY zeolite. This has been evidenced by the recycle use of these catalysts, as shown in Supplementary Fig. 14. After the first recycle use, a significant activity loss was observed with De-Al-HY zeolite. The glyceraldehyde conversion decreased from 93% to 50% in the second run and remained similar after five recycle uses. In contrast, nearly no activity loss could be observed with SA/50 after five recycle uses. This indicates that the Al species generated on the ASA catalysts are highly stable compared to extra-framework Al species in De-Al-HY zeolite, which are easily leaching out.

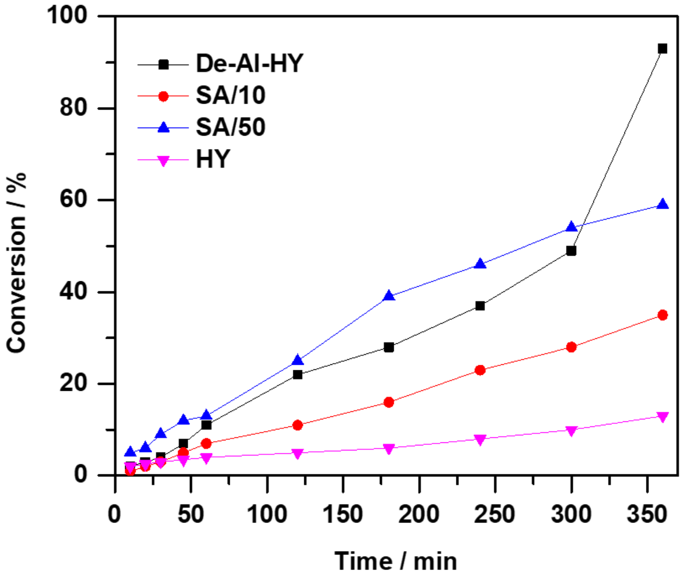

Supplementary Figure 13. Catalytic conversion of glyceraldehyde in ethanol over De-Al-HY (square), SA/10 (circle), SA/50 (up triangle), and HY (down triangle), as a function of time. Conditions: 1.25 mL aqueous ethanol (water/ethanol = 1:1 in volume ratio) solution containing 0.25 M glyceraldehyde, 0.05 g catalyst, at 363 K for 6 h with stirring. Dealuminated zeolite De-Al-HY (Si/Al = 5.4) was obtained by steaming zeolite H-Y (Si/Al = 2.7) at 748 K for 2.5 h.[14]

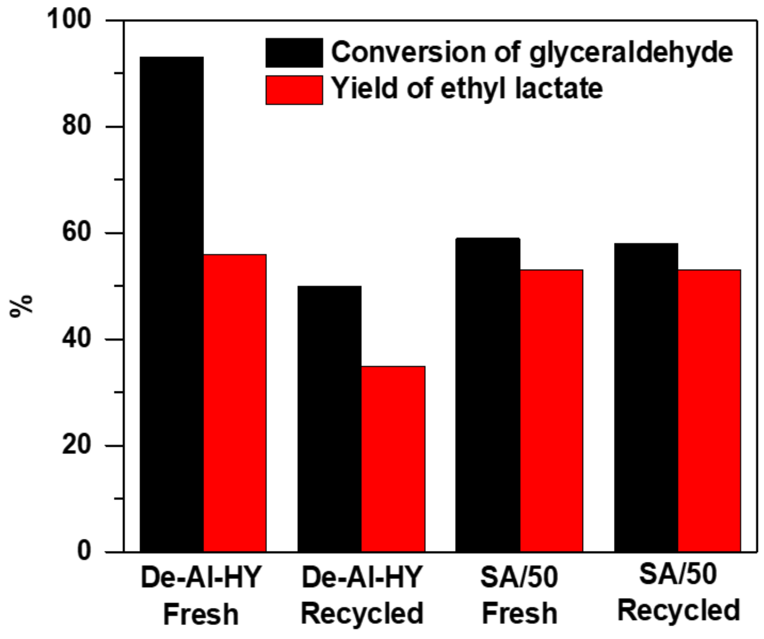

Supplementary Figure 14. Catalytic conversion of glyceraldehyde in ethanol over fresh and recycled De-Al-HY and SA/50. Conditions are the same as shown in Supplementary Fig. 13. After each run, the reaction solution was removed, followed by washing the catalysts with ethanol (5 mL) and separating by centrifugation. The washing step was repeated 3 times to obtain the recycled catalysts.

Supplementary Note 8. ^{27}Al 1D spectra of dealuminated zeolite HY

Two signals at 60 and 5 ppm with a hump at ca. 35 ppm can be observed in Supplementary Fig. 15. The signal at 60 ppm can be assigned to framework Al^{IV}, which can contribute to the formation of BAS via bridging OH groups in zeolites. The other two signals are attributed to the extra-framework species, which can afford the surface Lewis acidity. The extra-framework Al species decreased around 10 mol% after reaction. This indicates that the extra-framework Al species can be leached out during liquid-phase reaction, which was also confirmed by Al species detected in the reaction mixture.

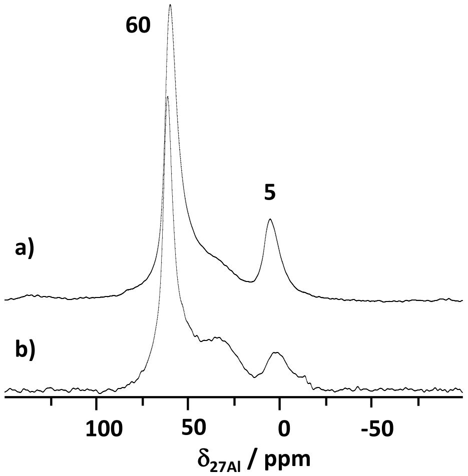

Supplementary Figure 15. ^{27}Al single-pulse MAS spectra recorded at 9.4 T with ν_R = 8 kHz for De-Al-HY: (a) fresh; (b) catalyst recycled after the catalytic conversion of glyceraldehyde in ethanol, followed by calcination at 773 K in air to remove organics. De-Al-HY zeolite was prepared as described in the caption of Supplementary Fig. 13.

Supplementary References

[1] Cory, D.G. and Ritchey, W.M. Suppression of signals from the probe in bloch decay spectra. *J. Magn. Reson.* **80,** 128-132 (1988).

[2] Keller, T.C. *et al.* Synthesis-property-performance relationships of amorphous silica-alumina catalysts for the production of methylenedianiline and higher homologues. *J. Catal.* **344,** 757-767 (2016).

[3] Huang, J., van Vegten, N., Jiang, Y., Hunger, M., Baiker, A. Increasing the Bronsted acidity of flame-derived silica/alumina up to zeolitic strength. *Angew. Chem. Int. Ed.* **49,** 7776-7781 (2010).

[4] De Geuser, F.; Lefebvre, W.; Blavette, D. 3D atom probe study of solute atoms clustering during natural ageing and pre-ageing of an Al-Mg-Si alloy. *Philos. Mag. Lett.* **86,** 227-234 (2006).

[5] Haley, D.; Petersen, T.; Barton, G.; Ringer, S. P. Influence of field evaporation on Radial Distribution Functions in Atom Probe Tomography. *Philos. Mag.* **89,** 925-943 (2009).

[6] Otomo, R., Tatsumi, T., Yokoi, T. Beta zeolite: a universally applicable catalyst for the conversion of various types of saccharides into furfurals. *Catal. Sci. Techn.* **5,** 4001-4007 (2015).

[7] Corma, A., Iborra, S., Velty, A. Chemical routes for the transformation of biomass into chemicals. *Chemical Reviews* **107,** 2411-2502 (2007).

[8] Moreno-Recio, M., Santamaria-Gonzalez, J., Maireles-Torres, P. Bronsted and Lewis acid ZSM-5 zeolites for the catalytic dehydration of glucose into 5-hydroxymethylfurfural. *Chem. Eng. J.* **303,** 22-30 (2016).

[9] Jimenez-Morales, I., Moreno-Recio, M., Santamaria-Gonzalez, J., Maireles-Torres, P., Jimenez-Lopez, A. Production of 5-hydroxymethylfurfural from glucose using aluminium doped MCM-41 silica as acid catalyst. *Appl. Catal. B-Environ.* **164,** 70-76 (2015).

[10] Wang, Z. C. *et al.* Bronsted acid sites based on penta-coordinated aluminum species. *Nat. Commun.* **7,** 13820 (2016).

[11] Lang, S., Benz, M., Obenaus, U., Himmelmann, R., Hunger, M. Novel approach for the characterization of Lewis acidic solid catalysts by solid-state NMR spectroscopy. *ChemCatChem* **8,** 2031-2036 (2016).

[12] Wang, Z., Jiang, Y., Baiker, A., Huang, J. Efficient acid-catalyzed conversion of phenylglyoxal to mandelates on flame-derived silica/alumina. *ACS Catal.* **3,** 1573-1577 (2013).

[13] Pescarmona, P.P. *et al.* Zeolite-catalysed conversion of C(3) sugars to alkyl lactates. *Green Chem.* **12,** 1083-1089 (2010).

[14] Huang, J., Jiang, Y.J., Marthala, V.R.R., Ooi, Y.S., Hunger, M. Regioselective H/D exchange at the side-chain of ethylbenzene on dealuminated zeolite H-Y studied by in situ MAS NMR-UV/Vis spectroscopy. *ChemPhysChem* **9,** 1107-1109 (2008).
