# Supplementary material for: Acidity enhancement through synergy of penta- and tetra-coordinated aluminum species in amorphous silica networks
Source: Nat Commun. 2020 Jan 13;11:225. doi: 10.1038/s41467-019-13907-7 (PMC6957685; doi:10.1038/s41467-019-13907-7)
Supplement: Supplementary file 2 — Description of Additional Supplementary Files [file 41467_2019_13907_MOESM2_ESM.pdf]

Description of Additional Supplementary Files

File Name: Supplementary Movie 1
Description: APT video of SA/10

File Name: Supplementary Movie 2
Description: APT video of SA/50
